# Establishment of Fruit Bat Cells (*Rousettus aegyptiacus*) as a Model System for the Investigation of Filoviral Infection

**DOI:** 10.1371/journal.pntd.0000802

**Published:** 2010-08-24

**Authors:** Verena Krähling, Olga Dolnik, Larissa Kolesnikova, Jonas Schmidt-Chanasit, Ingo Jordan, Volker Sandig, Stephan Günther, Stephan Becker

**Affiliations:** 1 Institut für Virologie, Philipps-Universität Marburg, Marburg, Germany; 2 Institut für Virologie, Bernhard-Nocht-Institut für Tropenmedizin, Hamburg, Germany; 3 ProBioGen AG, Berlin, Germany; University of Texas Medical Branch at Galveston, United States of America

## Abstract

**Background:**

The fruit bat species *Rousettus aegyptiacus* was identified as a potential reservoir for the highly pathogenic filovirus Marburg virus. To establish a basis for a molecular understanding of the biology of filoviruses in the reservoir host, we have adapted a set of molecular tools for investigation of filovirus replication in a recently developed cell line, R06E, derived from the species *Rousettus aegyptiacus*.

**Methodology/Principal Findings:**

Upon infection with Ebola or Marburg viruses, R06E cells produced viral titers comparable to VeroE6 cells, as shown by TCID_50_ analysis. Electron microscopic analysis of infected cells revealed morphological signs of filovirus infection as described for human- and monkey-derived cell lines. Using R06E cells, we detected an unusually high amount of intracellular viral proteins, which correlated with the accumulation of high numbers of filoviral nucleocapsids in the cytoplasm. We established protocols to produce Marburg infectious virus-like particles from R06E cells, which were then used to infect naïve target cells to investigate primary transcription. This was not possible with other cell lines previously tested. Moreover, we established protocols to reliably rescue recombinant Marburg viruses from R06E cells.

**Conclusion/Significance:**

These data indicated that R06E cells are highly suitable to investigate the biology of filoviruses in cells derived from their presumed reservoir.

## Author Summary

Marburg virus and several species of Ebola virus are endemic in central Africa and cause sporadic outbreaks in this region with mortality rates of up to 90%. So far, there is no vaccination or therapy available to protect people at risk in these regions. Recently, different fruit bats have been identified as potential reservoirs. One of them is *Rousettus aegyptiacus*. It seems that within huge bat populations only relatively small numbers are positive for filovirus-specific antibodies or filoviral RNA, a phenomenon that is currently not understood. As a first step towards understanding the biology of filoviruses in bats, we sought to establish a model system to investigate filovirus replication in cells derived from their natural reservoir. Here, we provide the first insights into this topic by monitoring filovirus infection of a *Rousettus aegyptiacus* derived cell line, R06E. We were able to show that filoviruses propagate well in R06E cells, which can, therefore, be used to investigate replication and transcription of filovirus RNA and to very efficiently perform rescue of recombinant Marburg virus using reverse genetics. These results emphasize the suitability of the newly established bat cell line for filovirus research.

## Introduction

Bats have been shown to be hosts for many pathogens, including those causing tropical diseases, such as leptospira, Hendra virus, Nipah virus and SARS-like coronavirus [1]–[6]. In some cases the development of disease in humans has been directly linked to contact with infected bats. Recently, several species of fruit bats were identified as probable reservoirs for the filoviruses Marburg virus (MARV) and Ebola virus (EBOV) [7]–[11]. Filoviruses cause a severe hemorrhagic fever with case fatality rates of up to 90%, for which there is neither an approved vaccine nor specific treatment currently available [12]. As a result of this, as well as the fact that filoviruses represent a serious hazard for laboratory workers, they are classified as biosafety level 4 (BSL4) agents. The filovirus outbreaks in Central Africa occur sporadically and unpredictably, the latter contributes considerably to the public awareness of filovirus outbreaks. For more than 40 years the search for the natural reservoir of filoviruses was one of the most interesting endeavours in the field of highly pathogenic agents, and was fuelled by the dramatic outbreaks, cases of filovirus infected tourists and concerns that filoviruses might be abused as biological weapons.

In the case of EBOV, outbreaks could frequently be traced back to the preparation of bush meat, often from sick monkeys, for consumption [13], [14]. Since filovirus infection of monkeys results in a rapid and fatal hemorrhagic fever, it was considered that monkeys do not represent the natural reservoir of EBOV or MARV. Right from the beginning of the recorded history of filovirus outbreaks, the MARV outbreak in 1967, it was suspected that bats might also be connected to the spread of infection. This was emphasized by the observation that, in those cases where the consumption of contaminated bush meat could be ruled out as the source of infection, often a close contact between index cases and bats was observed [15]. In 1996, Swanepoel *et al.* were able to show that certain species of bats could be productively infected with EBOV without showing signs of disease, which was considered a prerequisite for serving as natural hosts [16]. Supporting this hypothesis, filoviral genomic RNA and antibodies could be detected in bats of different species from the region where outbreaks had occurred, providing the first evidence that bats are infected in a natural context [8], [11]. Finally, while MARV was isolated from samples of the Megachiropteran *Rousettus aegyptiacus* that were trapped in regions where outbreaks took place [15] the assumption that this fruit bat species can also serve as a reservoir for EBOV is based on serologic data [9].

The filoviruses EBOV and MARV are enveloped RNA viruses with a filamentous shape and constitute the family *Filoviridae* within the order *Mononegavirales*. The family of *Filoviridae* contains the genera *Marburgvirus* and *Ebolavirus* [12]. Filoviruses contain a non-segmented negative-strand 19 kb RNA genome, which encodes seven structural proteins and an additional nonstructural protein in the case of Ebola virus. The genome is associated with four nucleocapsid proteins: NP, VP30, VP35 and L [17]. NP encapsidates the viral genome and is, together with the polymerase L and the polymerase cofactor VP35, necessary and sufficient for viral replication. VP30, the fourth nucleocapsid protein, represents an essential transcription factor for Ebola virus [18]–[20]. The filoviral nucleocapsid is enclosed by two matrix proteins, VP40 and VP24 that connect the nucleocapsid with the lipid envelope [17]. The transmembrane glycoprotein GP is inserted in the envelope, where it recognizes target cells and induces fusion between cellular and viral membranes [21]–[23].

So far, little is known about the filoviral life cycle in the presumed reservoir. One study describes persistent infection of a Mexican free-tailed bat cell line Tb1.Lu with EBOV. The authors showed that EBOV replication in these cells was low but could be stimulated by inducing the Ras/MAPK pathway [24]. The Mexican free-tailed bat belongs to the order Microchiroptera and is abundant in North America but is only very distantly related to Megachiroptera such as *Rousettus aegyptiacus*, the presumed natural reservoir of filoviruses. The unavailability of a cell line from a bat species that is relevant for filovirus transmission to humans constrains research of filovirus biology in the natural reservoir. Recently, Jordan *et al.* presented a newly established cell line derived from *Rousettus aegyptiacus* (R06E), which could presumably close this gap by allowing *in vitro* studies to understand the replication of filoviruses in bats [25]. So far, filoviruses have been propagated in human or monkey cell lines and it was now of interest to determine whether cells from the natural host replicate filoviruses, and if so to characterize the infection. Here we have examined whether the R06E cell line is suitable for investigations of filovirus infection.

## Methods

### Cell culture and virus infection

VeroE6 (African green monkey kidney cells), HEK293 (human embryonic kidney cells), HUH7 (human hepatoma cells) and R06E cells (derived from *Rousettus aegyptiacus*) [25] were cultured in Dulbecco's modified Eagle medium (DMEM) supplemented with 10% fetal calf serum (FCS), penicillin (50 units/mL), and streptomycin (50 µg/mL). The Leiden strain of MARV, which was isolated in 2008 from a tourist visiting a bat-infested cave in Uganda [26], or the Mayinga strain of *Zaire ebolavirus* (ZEBOV) (GenBank accession number NC002549) were propagated in VeroE6 cells. Virus titers were determined by 50% tissue culture infectious dose (TCID_50_) assays. Cells were infected with MARV or ZEBOV with 0.1, 0.5 or 5 TCID_50_/ml per cell, as indicated. All work with filoviruses was performed in the biosafety level 4 (BSL4) facility of the Philipps University, Marburg.

### TCID_50_ assay

VeroE6 cells were cultured in 96-well plates to 50% confluence and infected with 10-fold serial dilutions of supernatants from infected cells. At 10 to 14 days post infection (p.i.), when the cytopathic effect had stabilized, cells were analyzed by light microscopy. The TCID_50_/ml was calculated using the Spearman-Kärber method [27].

### Electron microscopy

Infected cells were fixed 2 or 3 days p.i. in paraformaldehyde (PFA) and glutaraldehyde (4% PFA, 0.1% glutaraldehyde in 60 mM Pipes, 25 mM Hepes, 2 mM MgCl_2_, 10 mM EGTA, pH 7.0), scraped off after 30 minutes and centrifuged at 20,000×g for 20–30 minutes. The fixative solution was replaced with 4% PFA in DMEM to completely inactivate the sample overnight before removal from the BSL4 laboratory. Dehydration of infected cells and embedding in Epon were performed as described previously [28]. Ultrathin sections were stained with uranyl acetate and lead citrate and observed with a Zeiss 109 electron microscope. Supernatants of the infected cells were centrifuged through a 20% sucrose cushion at 77,000×g at 4°C to concentrate the viral particles. The pellet was inactivated and fixed with 4% PFA in DMEM overnight. Negative staining of viral suspensions was done with 2% phosphotungstic acid.

### Minigenome and iVLP assay

Cells were transfected with plasmids encoding a MARV minigenome (3M–5M-Luc, 1 µg) [20], [29], [30] and the viral proteins necessary for replication and transcription: NP (0.1 µg), VP35 (0.5 µg), VP30 (0.1 µg), and the polymerase L (1 µg) using TransIT-LT1 (Mirus, Madison, WI, USA) according to the manufacturer's instructions. The minigenome contained a renilla luciferase reporter gene instead of the CAT gene [30], [31]. In addition, a plasmid coding for the T7 polymerase (0.5 µg) and a construct constitutively expressing a firefly luciferase reporter (pGL4, Promega, 0.1 µg), suitable for normalization of transfection efficiency, were co-transfected. At 48 h post transfection (p.t.), cells were lysed in passive lysis buffer (Promega, Madison, WI, USA). Luciferase assays were performed using the Promega Dual-Luciferase reporter assay system. Relative firefly luciferase signals were used to normalize for transfection efficiency.

The MARV iVLP assay was performed as described by Wenigenrath *et al.* [30]. At 48 h p.t., cells and supernatants were harvested. iVLPs from the supernatant were used to infect cells that were either untreated or pretransfected with plasmids encoding the nucleocapsid proteins. These cells were analyzed 48 or 72 h p.i. for luciferase reporter activity.

Student's *t* test was performed to analyze statistical significances within the different *in vitro* assays.

### Western blot analysis

Whole-cell extracts were prepared by lysing cells in sodium docecyl sulfate (SDS) sample buffer (25% glycerol, 2.5% SDS, 125 mM Tris [pH 6.8], 125 mM dithiothreitol, 0.25% bromophenol blue). Samples were boiled for 10 minutes at 99°C and transferred into a fresh tube before removal from the BSL4 facility. Proteins were separated on 12% SDS polyacrylamide gels and transferred onto polyvinylidene difluoride membranes. Immunostaining was performed with dilutions of primary antibody in phosphate-buffered saline containing 1% (w/v) skim milk and 0.1% Tween-20, as indicated below. VP40-specific monoclonal antibodies (MARV: anti-VP40 40-2-2 (1∶2000); ZEBOV: anti-VP40 2C4 (1∶200)) were used to detect the VP40 proteins of the respective virus. A mouse monoclonal antibody was used to detect α-tubulin (Clone DM 1A, 1∶5000; Sigma-Aldrich, St.Louis, MO, USA) as a cellular control protein. Western blot detection was performed with Alexa 680-conjugated anti mouse immunoglobulin G secondary antibody using the Odyssey Infrared Imaging System (LI-COR, Lincoln, NE, USA).

The total amount of protein in the cell lysates was determined by separating cell lysates on 12% SDS polyacrylamide gels which were stained with Coomassie Brilliant Blue. Gels were then quantified by using the Odyssey Infrared Imaging System Application Software (Version 2.1.12).

### Rescue of recombinant Marburg virus

R06E cells were grown in 6-well plates to 50% confluence. Transfection of plasmids coding for the supporting nucleocapsid proteins NP (0.5 µg), VP35 (0.1 µg), VP30 (0.1 µg) and L (2 µg) as well as the T7 polymerase (0.5 µg) and the full-length cDNA construct of MARV (2 µg) was performed with TransIT-LT1 (Mirus, Madison, WI, USA) according to the manufacturer's instructions. Five hours p.t. the medium was replaced with 4 ml DMEM with 2.5% fetal calf serum. Cells and supernatants were harvested between day 6 and 12 after CPE formation. Supernatant was used to infect fresh R06E cells (passage 1). When passage 1 cells displayed a CPE, viral RNA was isolated from the supernatant using the QIAamp Viral RNA Mini Kit (QIAGEN, Venlo, Netherlands). One-twelfth of the eluted RNA was used for reverse transcription-PCR (RT-PCR) using the Transkriptor One-Step RT-PCR Kit (Roche, Basel, Switzerland) and glycoprotein-specific primers designed to amplify nt 5889 to 7370 of the MARV genome (forward: 5′-CAGGTCGACTCAGTGAATATATTCTCATAT-3′; reverse 5′-GAGGCACCAGAACTAGAGGA). Amplified fragments were restricted with *KpnI* to distinguish recombinant and wild type virus. Furthermore, integrity of recombinant viral RNA was verified by sequencing of the complete genome. Cells and supernatants of passage 1 were inactivated by addition of 1× SDS sample buffer and boiling for 10 min at 99°C. Samples were subjected to Western blot analysis with VP40- and NP-specific monoclonal antibodies (MARV: anti-VP40 40-2-2 (1∶2000), anti-NP 59-9 (1∶500); ZEBOV: anti-VP40 2C4 (1∶200)).

## Results and Discussion

**Figure 1:**
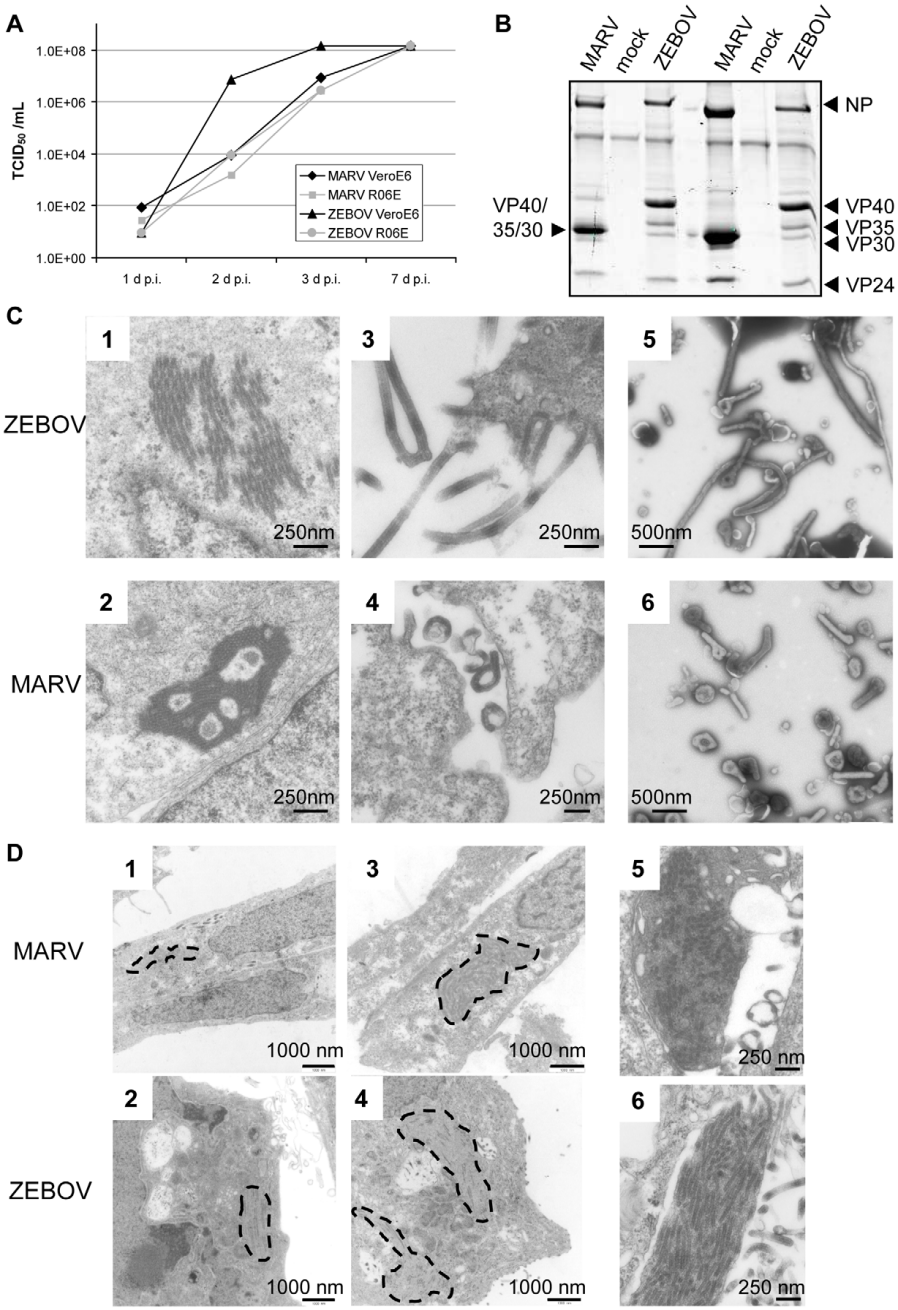
Infection of R06E cells with MARV and ZEBOV. (A) VeroE6 or R06E cells were infected with MARV or ZEBOV with 0.5 TCID_50_/cell. Supernatants were collected at 1, 2, 3 and 7 days p.i. and used for TCID_50_ assays. (B) Supernatant from day 7 was concentrated via ultracentrifugation and viral particles were analyzed by Coomassie staining for protein composition. (C) R06E cells were infected with MARV and ZEBOV at a high MOI, fixed and inactivated at day 3 p.i.. Cells were dehydrated and embedded in Epon prior to ultrathin sectioning. Analysis by transmission electron microscopy showed viral inclusions in the perinuclear region (1, 2) and mature viral particles (3, 4). MARV and ZEBOV particles were purified via ultracentrifugation through a 20% sucrose cushion, fixed with 4% paraformaldehyde, negatively stained and analyzed by electron microscopy (5, 6). (D) VeroE6 and R06E cells were infected with MARV and ZEBOV at a high MOI, harvested at 48 h p.i. and treated as described under B. Analysis by transmission electron microscopy showed viral inclusions (broken lines) in the perinuclear region of VeroE6 (1, 2) and R06E (3, 4) cells at low magnification. Higher magnification pictures of viral inclusions in R06E cells are shown under 5 and 6.

To determine whether R06E cells are susceptible to filovirus infections, we infected R06E and VeroE6 cells, which are commonly used to prepare stock virus [32]–[34], with MARV, strain Leiden [26], or ZEBOV, strain Mayinga using 0.5 TCID_50_ per cell. Both cell lines exhibited a strong cytopathic effect (CPE) at day 7 p.i.. Supernatants of infected cells collected at day 1, 2, 3 and 7 p.i. were subjected to TCID_50_ analysis, which revealed that MARV showed similar growth kinetics and end titers in R06E cells and VeroE6 cells. ZEBOV grew faster in VeroE6 cells than in R06E cells but reached the same maximum titer at 7 d p.i. (Fig. 1A). When the two cell lines were infected with 5 TCID_50_ per cell of EBOV or MARV no differences between R06E and VeroE6 cells were observed (not shown). Investigations to analyze the reason of the differences between high and low infectious doses are underway.

The protein composition of viral particles from the supernatants of both cell lines was analyzed by Coomassie Brilliant Blue staining. This analysis did not show significant differences in the viral protein composition between virus produced during infection of R06E cells and VeroE6 cells (Fig. 1B).

Next we investigated the structure of filovirus-infected R06E cells and viral particles, by electron microscopy (EM). Viral inclusions in perinuclear regions were composed of hexagonally arranged nucleocapsids with characteristic electron-transparent internucleocapsid zones for ZEBOV (Fig. 1C 1) and a higher density of nucleocapsid packaging for MARV (Fig. 1C 2). At the plasma membrane, mature viral particles in the process of budding or completely formed were readily observed (Fig. 1C 3 and 4). EM analysis of negatively stained samples of the supernatants of R06E cells infected with MARV or ZEBOV showed typical filamentous particles (Fig. 1C, 5 and 6). Notably, the number of intracellular MARV and EBOV nucleocapsids in R06E cells was considerably higher than in VeroE6 cells. This is reflected by the larger size of viral inclusions, which represent accumulations of viral nucleocapsids in R06E (Fig. 1D 3–6) compared to VeroE6 cells (Fig. 1D 1,2). We were, therefore, interested to further investigate whether the amount of intracellular viral proteins was different in VeroE6, HUH7 and R06E cells.

**Figure 2:**
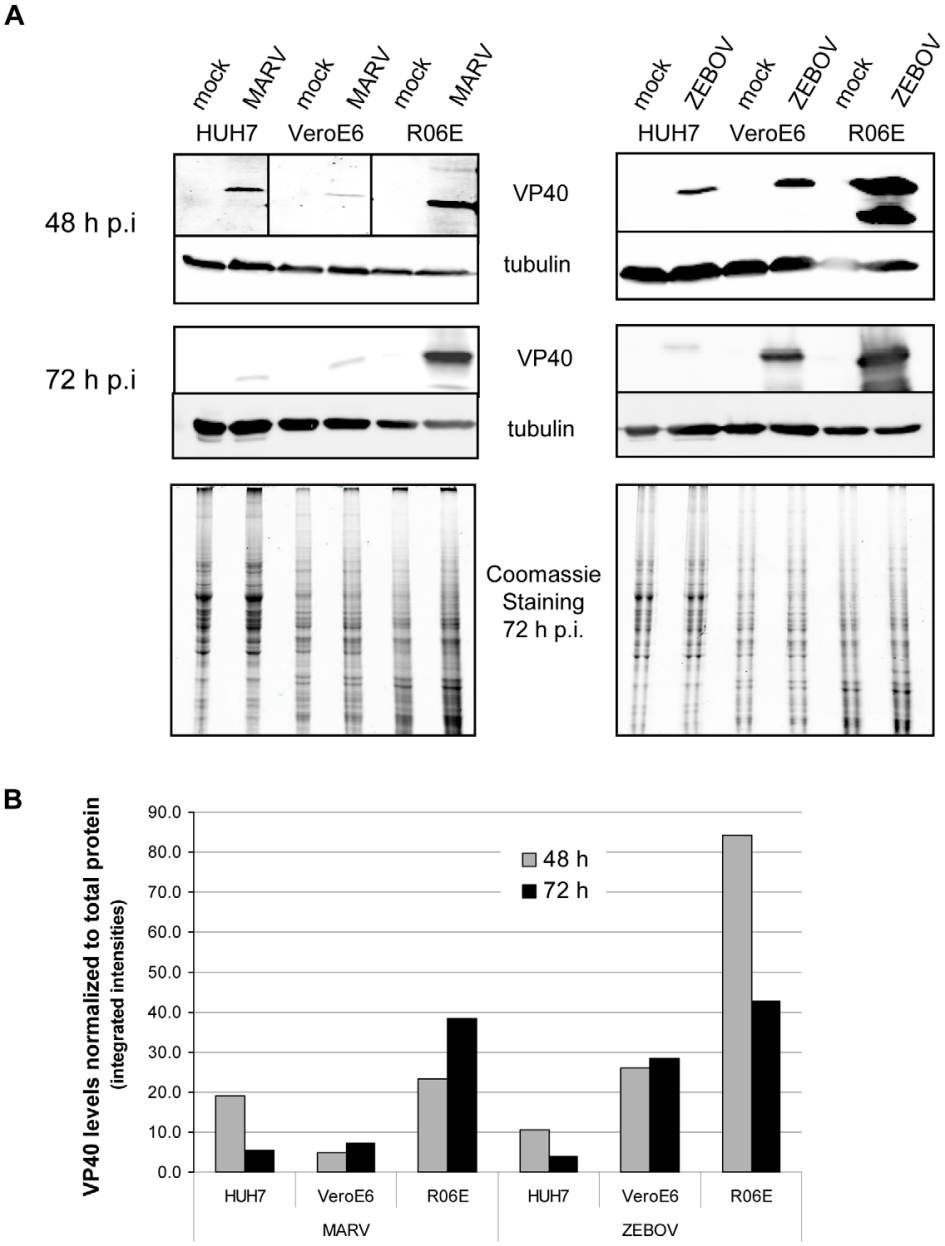
Expression of viral proteins in different cell lines. (A) 4×10^5^ HUH7, VeroE6 or R06E cells were infected with 0.1 TCID_50_/cell MARV or ZEBOV. Cell lysates were prepared at 48 and 72 h p.i., subjected to Western blot analysis to detect cellular tubulin and VP40 of MARV and ZEBOV using mouse monoclonal antibodies. The total amount of cellular proteins in the samples was quantified by separating cell lysates on SDS PAGE, which were then stained with Coomassie Blue. Using the Odyssey Infrared Imaging Application Software, the protein signals were quantified. VP40 levels normalized to total cell protein are shown in (B).

The three cell lines were infected with 0.1 TCID_50_ per cell and Western blot analysis was performed with lysed cells at 48 and 72 h p.i. using monoclonal antibodies specific for MARV or ZEBOV VP40 or the cellular cytoskeleton protein tubulin. These analyses revealed that the fruit bat cells accumulated considerably more viral protein than the other cell lines tested (Fig. 2A). To further support this result, we quantified total protein levels of the different cell lines by Coomassie staining of cell lysates separated by SDS gels at 48 h and 72 h p.i. which were scanned using the Odyssey Analyzer (The 72 h gels are shown beneath the Western blots in Fig. 2A) The amount of VP40 was then normalized against the total cell protein (Fig. 2B). Quantification clearly shows that at 48 as well as 72 h p.i. the highest amount of viral protein can be found in R06E cells when compared to HUH7 or VeroE6 cells (Fig. 2B). However, our data also indicate that R06E cells support filoviral propagation to an extent comparable with VeroE6 or HUH7 cell lines. Furthermore, EM analysis of ultrathin sections of R06E cells revealed morphological signs of filoviral infection similar to those detected previously in cells originating from monkeys or humans [35], [36] (Fig. 1C). Taken together, these observations suggest that R06E cells may not release viral particles with a comparable efficiency to that with which they produce structural proteins. Further experiments to examine the viral budding efficiency and interaction of viral proteins with proteins of the endosomal sorting complex required for transport (ESCRT) in fruit bat-derived cells will be very important, as it has been shown that interaction of MARV VP40 with TSG101 or interaction of ZEBOV VP40 with TSG101 and Nedd4 plays an important role during the budding process [37]–[39].

**Figure 3:**
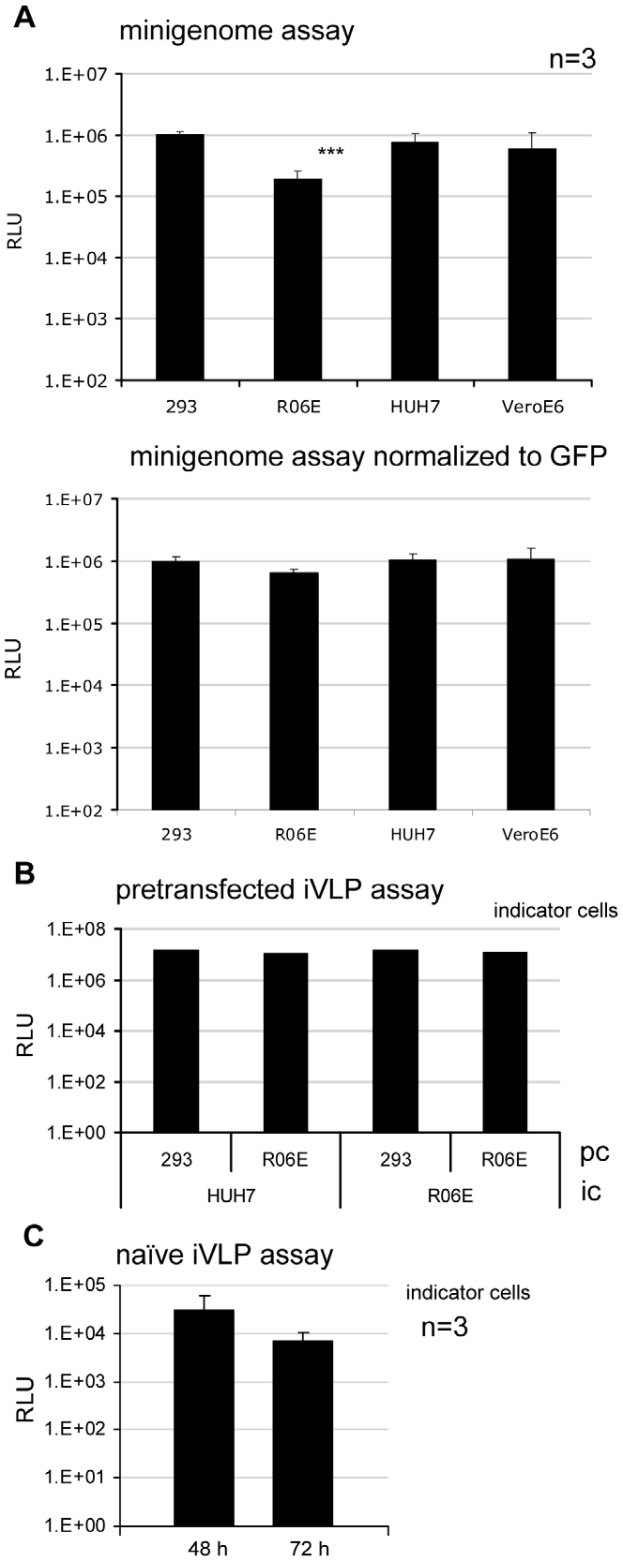
MARV-specific *in vitro* assays. (A) Minigenome assay. Different cell lines were transfected with all of the plasmids necessary for replication and transcription of a MARV (3M–5M) minigenome. Relative light units (RLU) represent the efficiency of replication and transcription of the minigenome (upper panel). This experiment was performed in triplicate and standard deviations are shown. Asterisks indicate statistically significant differences (*** P-value≤0.002) RLUs shown in the upper panel were normalized to the transfection efficiency of the cells, as analyzed by a GFP-reporter construct. (lower panel) (B) iVLP assay with pretransfected indicator cells. HEK293 or R06E cells were transfected with all of the plasmids necessary to produce MARV iVLPs (producer cells, pc). Supernatants were collected 72 h p.t. and used to infect new indicator cells (HUH7 or R06E cells) pretransfected with all of the plasmids necessary for replication and transcription (indicator cells, ic). Three days p.i. the luciferase activity in the ic was determined, reflecting iVLP formation, budding, iVLP entry, minigenome delivery and secondary transcription. (C) iVLP assay with naïve indicator cells. R06E cells were transfected with all of the plasmids necessary to produce iVLPs (pc). Supernatant was collected 72 h p.t. and used to infect R06E cells that were not pretransfected. Luciferase activity in the indicator cells was determined 48 or 72 h p.i., reflecting iVLP formation, budding, iVLP entry, minigenome delivery and primary transcription of minigenomes. This experiment was performed in triplicate and standard deviations are shown.

To further investigate the suitability of R06E cells for the analysis of filovirus replication and transcription we used artificial filovirus minigenome systems [20], [29]. Four different cell lines, R06E, HEK293, HUH7 and VeroE6 were transfected with plasmids encoding a MARV minigenome and the viral proteins necessary for replication and transcription (NP, VP35, VP30 and L). Cell lysates were analyzed 48 h p.t. for luciferase reporter activity. All analyzed cell lines showed nearly the same reporter activity, except for the fruit bat cell line where activity was slightly decreased (Fig. 3A, upper panel). Using a plasmid encoding the green fluorescence protein (GFP) for transfection of cells we found that the transfection efficiency was 15% for R06E cells, 50% for HEK293, 38% for HUH7 and 27% for VeroE6 cells (data not shown). When the transfection efficiency was taken into account, reporter signal in all tested cell lines showed no significant differences (Fig. 3A, lower panel). Furthermore we have tested the Ebola virus minigenome system with R06E, HUH7, VeroE6 and 293 cells and got comparable reporter gene activity (data not shown).

It was then investigated whether it was possible to produce MARV infectious virus-like particles (iVLPs) in R06E cells [30] and to establish a naïve iVLP assay for MARV to investigate primary transcription, as has already been described for ZEBOV [31]. R06E or HEK293 cells (producer cells, pc) were transfected with plasmids encoding all MARV structural proteins, the minigenome and the T7 support plasmid [30]. At 48 h p.t., cells and supernatants were harvested and iVLPs from the supernatant were used to infect HUH7 or R06E cells (indicator cells, ic), which were either untreated or pretransfected with plasmids encoding the nucleocapsid proteins. We did not detect a significant difference in the luciferase reporter signal in ic, when these were pretransfected (Fig. 3B). Interestingly, when non-transfected ic (naïve) were infected with iVLPs, only R06E cells showed reporter gene signals (Fig. 3C). Further experiments with the naïve iVLP assay revealed that using HEK293 cells to produce the iVLPs (pc) and R06E cells as ic produced the highest reporter gene signals in the indicator cells (data not shown). This combination took advantage of the very high transfection efficiency of HEK293 cells for iVLP production and the high capacity for efficient production of viral proteins in R06E cells.

**Figure 4:**
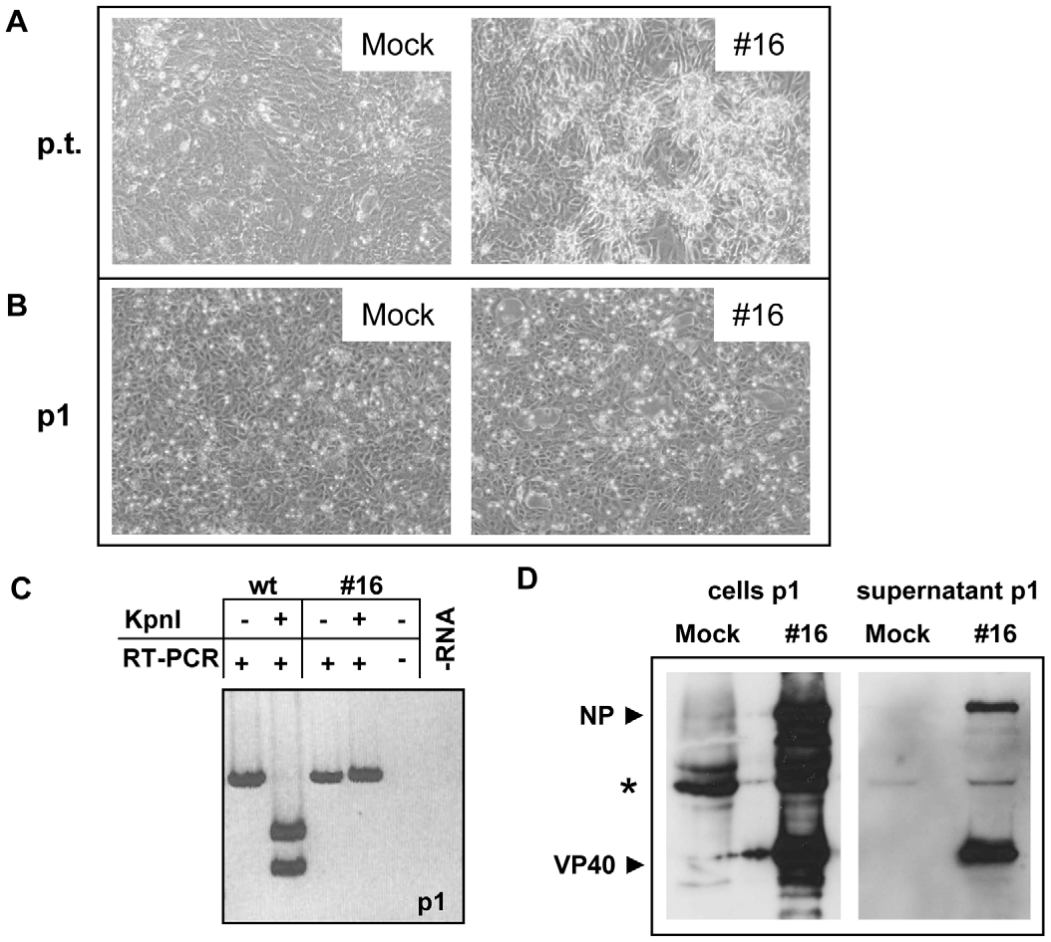
Rescue of recombinant MARV from R06E cells. R06E cells were transfected with all the plasmids sufficient for replication and transcription, a T7 polymerase construct and a T7-driven full-length cDNA construct of MARV (clone #16). (A) CPE formation was monitored at day 7 post transfection (p.t.). (B) Supernatant of transfected cells collected on day 7 was used to infect fresh VeroE6 cells (passage 1, p1). CPE formation was monitored 8 days post infection. (C) Supernatant of p1 was collected on day 8 p.i. and viral RNA was extracted. Glycoprotein gene-specific RT-PCR and subsequent restriction of the DNA with *KpnI*, a restriction site present only in the wild type genome, revealed the rescue of recombinant virus. (D) On day 8 p.i. supernatant and cells were lysed and subjected to Western blot analysis using monoclonal antibodies to detect the MARV proteins NP and VP40. * unknown cellular protein.

It was then investigated whether recombinant MARV could be rescued from R06E cells. Previous attempts using the experimental set-up described by Enterlein *et al.* had not worked in our hands [40]. Finally, after having unsuccessfully tested several combinations of cell lines for transfection and passage of potentially rescued virus, we used R06E cells for transfection of plasmids encoding the MARV nucleocapsid proteins as well as the T7 polymerase together with the plasmid containing the full length genome of MARV under the control of the T7 promoter. After 6–12 days cells and supernatant were harvested. The supernatant was used to infect fresh R06E cells (passage 1). This method allowed us to rescue three different recombinant viruses of which rescue of clone #16 is shown in Fig. 4. In the transfected cells, CPE formation was visible at day 7 p.t. and at day 8 after passaging the supernatant to fresh cells. At this time, RNA was purified from supernatant and RT-PCR was performed with GP-specific primers. The amplified fragments were restricted with *KpnI*, the restriction site for which was mutated in the recombinant full-length genome. Agarose gel electrophoresis of the fragments showed that, while control PCR fragments derived from the wild-type MARV genome were restricted by *KpnI*, fragments from clone #16 were not, indicating that clone #16 was a recombinant virus. In addition, Western blot analyses with VP40- and NP-specific monoclonal antibodies were performed to confirm the presence of recombinant virus in the supernatant. Taken together, our experiments suggest that the efficient rescue in R06E cells is the result of an exceptional support of MARV replication in the fruit bat cell line. This observation is consistent with the highly efficient expression of MARV proteins in R06E cells and their ability to release high titers of MARV in the supernatant.

At first glance the high expression levels of viral proteins and high viral titers found in the supernatants of the fruit bat-derived cell line R06E are puzzling in light of the presumption that *Rousettus aegyptiacus* is a reservoir for filoviruses. However, experimental infection of fruit bats with ZEBOV has been shown to result in productive infection and high titers of virus without clear signs of illness in the infected animals [16]. A more recent study described a similar observation in the reservoir fruit bat of Nipah virus. In this study Nipah virus-infected *Pteropus* bats developed a subclinical infection, neutralizing antibodies were raised in the serum of all animals and virus was excreted periodically [41]. While the mechanisms that result in persistence of filoviruses in the natural host are not understood, it is possible that the huge amounts of viral protein produced by primary target cells induce a rapid immune response leading to clearance of the virus from circulation, with the exception of tissues that are less accessible to the immune response. For filoviruses it has been shown that in the course of human infection, infectious virus continues to be detected in the semen for more than 80 days after infection and can lead to sexually transmitted disease as was the case for Marburg virus [42], [43]. It is therefore of interest to investigate whether hidden repositories of virus are present within infected *Rousettus aegyptiacus* that are then reactivated under certain unknown conditions. In addition, using the R06E cells, it will be of great interest to investigate how the bats interferon system responds to filovirus infection. Studies are underway to explore whether filoviruses are able to induce a persistent infection in R06E cells and to further analyze the innate immune response of the cells to infection with filoviruses.

In summary, we were able to show that R06E cells can be infected productively with filoviruses, giving rise to infectious viral particles. Infected cells show morphological signs of filoviral infection similar to those detected previously in cells originating from monkeys or humans. Further, R06E cells seem to be more effective in producing recombinant MARV than other cell lines tested and allowed for the establishment of a naïve infectious VLP assay for MARV. These results emphasize the suitability of the newly established bat cell line for further research in numerous areas of filovirus biology.
